# Supplementary material for: Climate change and land use threaten global hotspots of phylogenetic endemism for trees
Source: Nat Commun. 2023 Oct 31;14:6950. doi: 10.1038/s41467-023-42671-y (PMC10618213; doi:10.1038/s41467-023-42671-y)
Supplement: Supplementary file 1 — Supplementary information [file 41467_2023_42671_MOESM1_ESM.pdf]

Supporting information

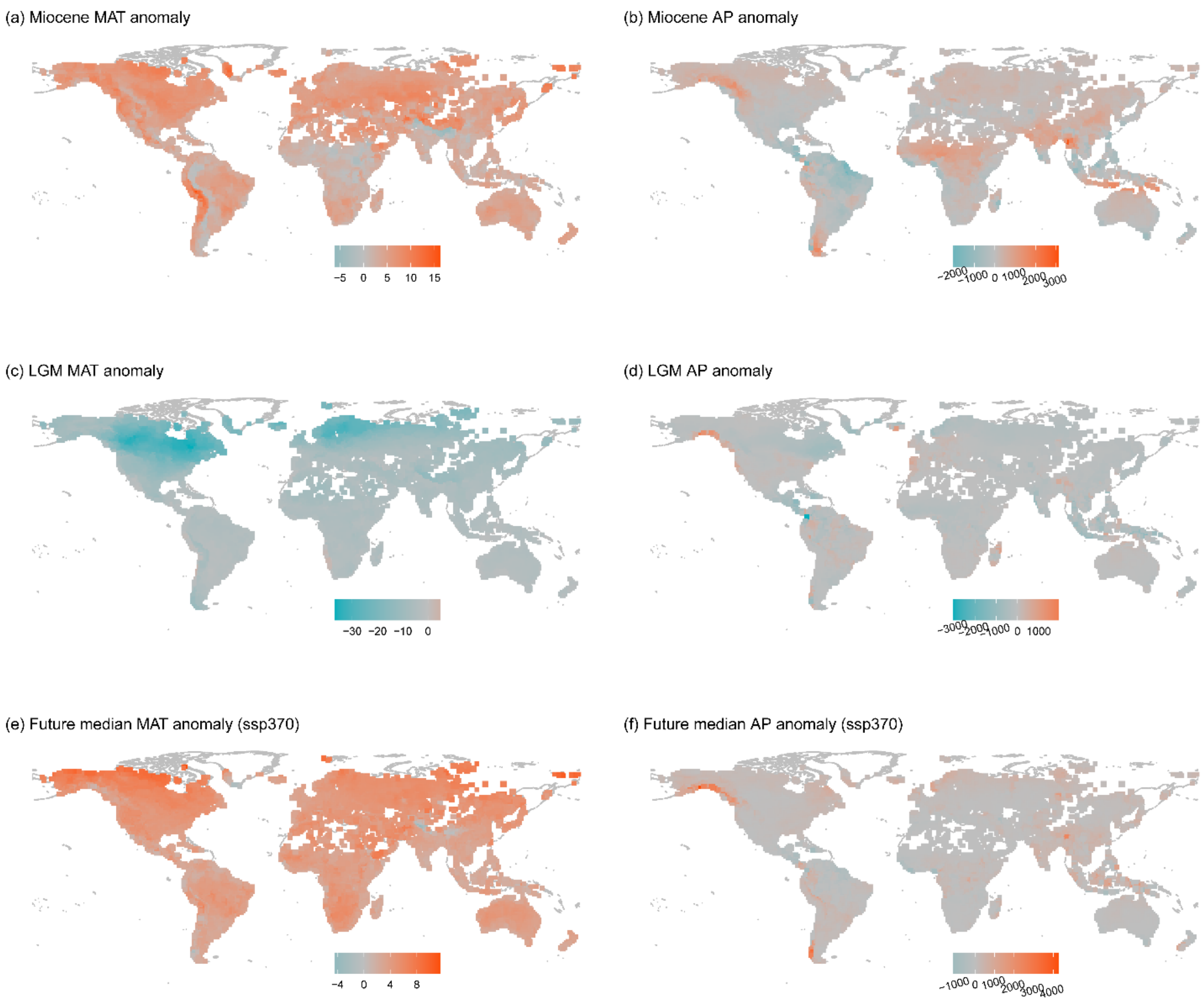

Fig. S1 Maps of the MAT (°C) and AP (mm) anomaly in each paleo-period and future (2070). LGM, Last Glacial Maximum; MAT, mean annual temperature; AP, annual precipitation. Anomaly is calculated as the difference between the paleo-/future climate variable and the corresponding current climate variable (i.e., LGM MAT – current MAT). LGM: Last Glacial Maximum.

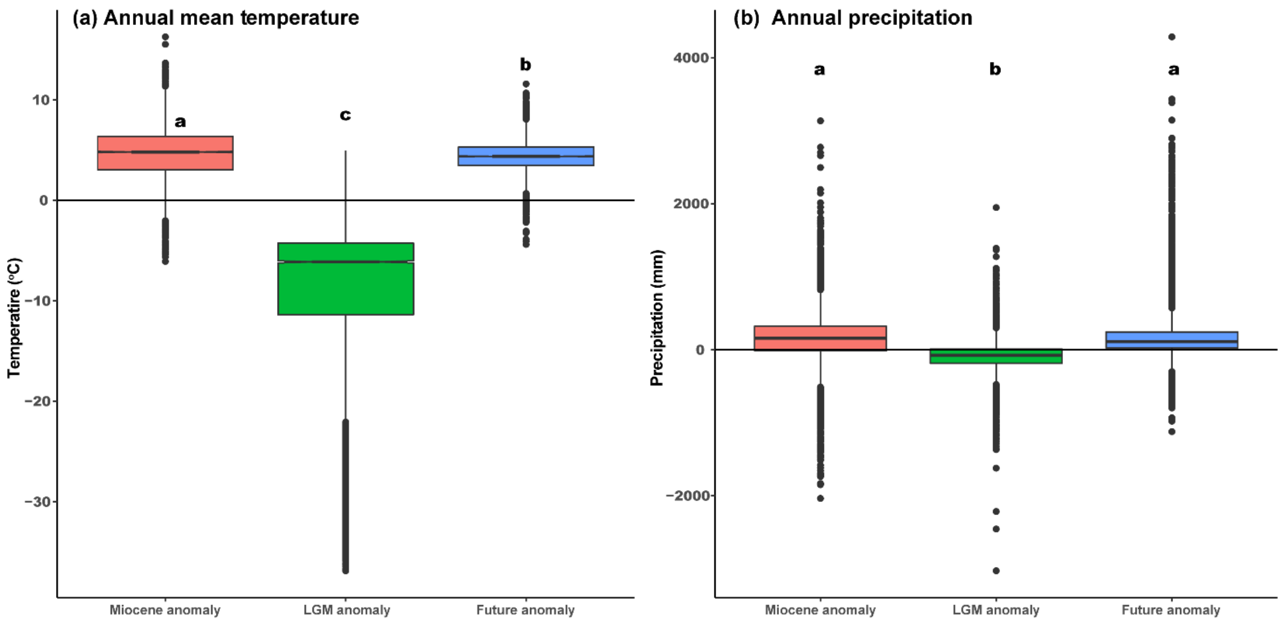

Fig. S2 Boxplots of the temperature anomaly (a) and precipitation anomaly (b). Anomaly was calculated as the difference between the paleo-/future (2070) climate variable and the corresponding current climate variable (i.e., LGM MAT – current MAT). LGM: Last Glacial Maximum.

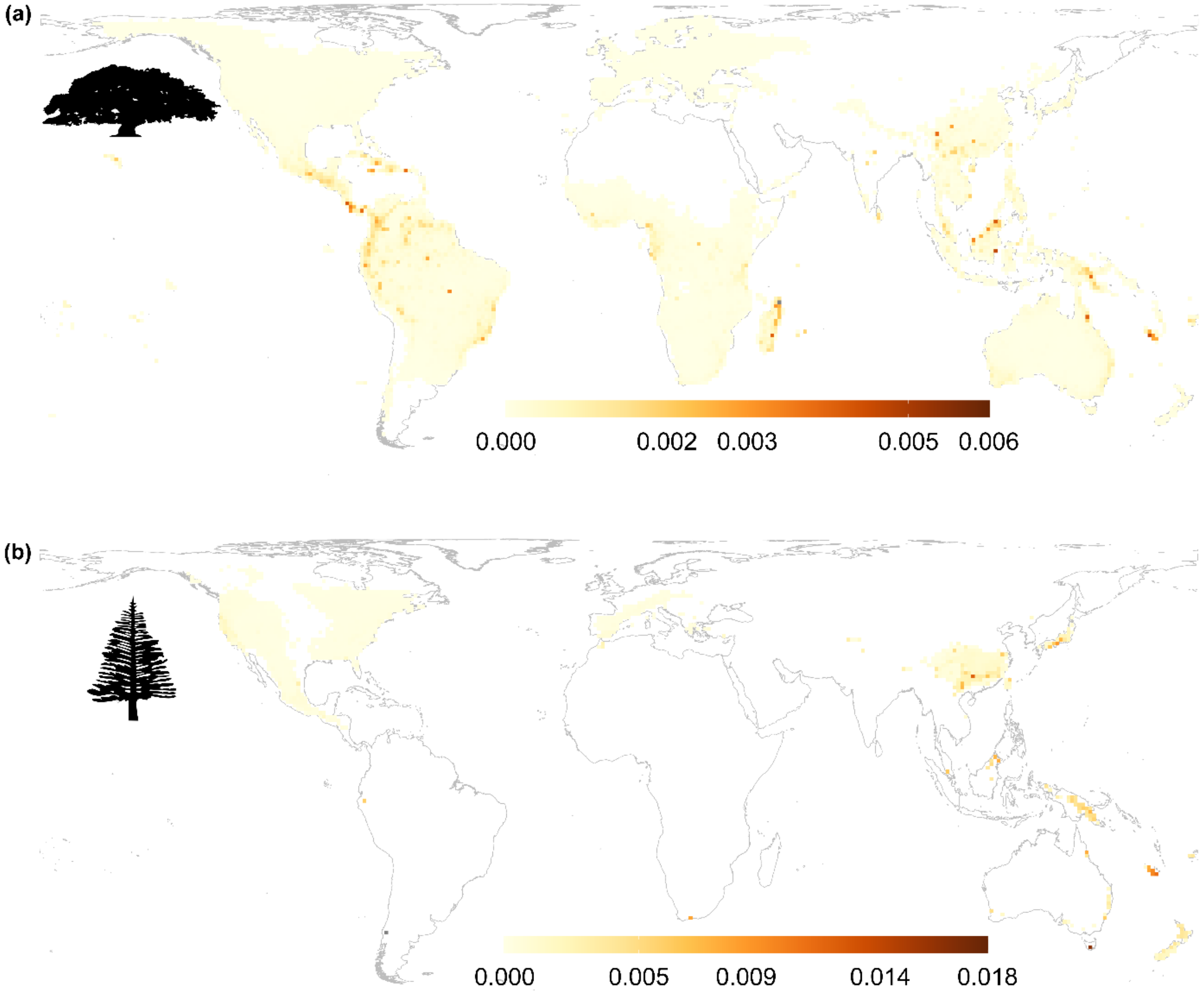

Fig. S3 Observed phylogenetic endemism for (a) angiosperm and (b) gymnosperm trees. The difference between this figure and Fig. 1 was that the global maps here were not masked by coastlines; in contrast, Fig. 1 represents only cells with more than 50% of the areas within landline, thus some grid cells, such as New Caledonia, were discarded. Pictograms courtesy of PhyloPic (www.phylopic.org): (a) Tracy A. Heath; (b) T. Michael Keesey.

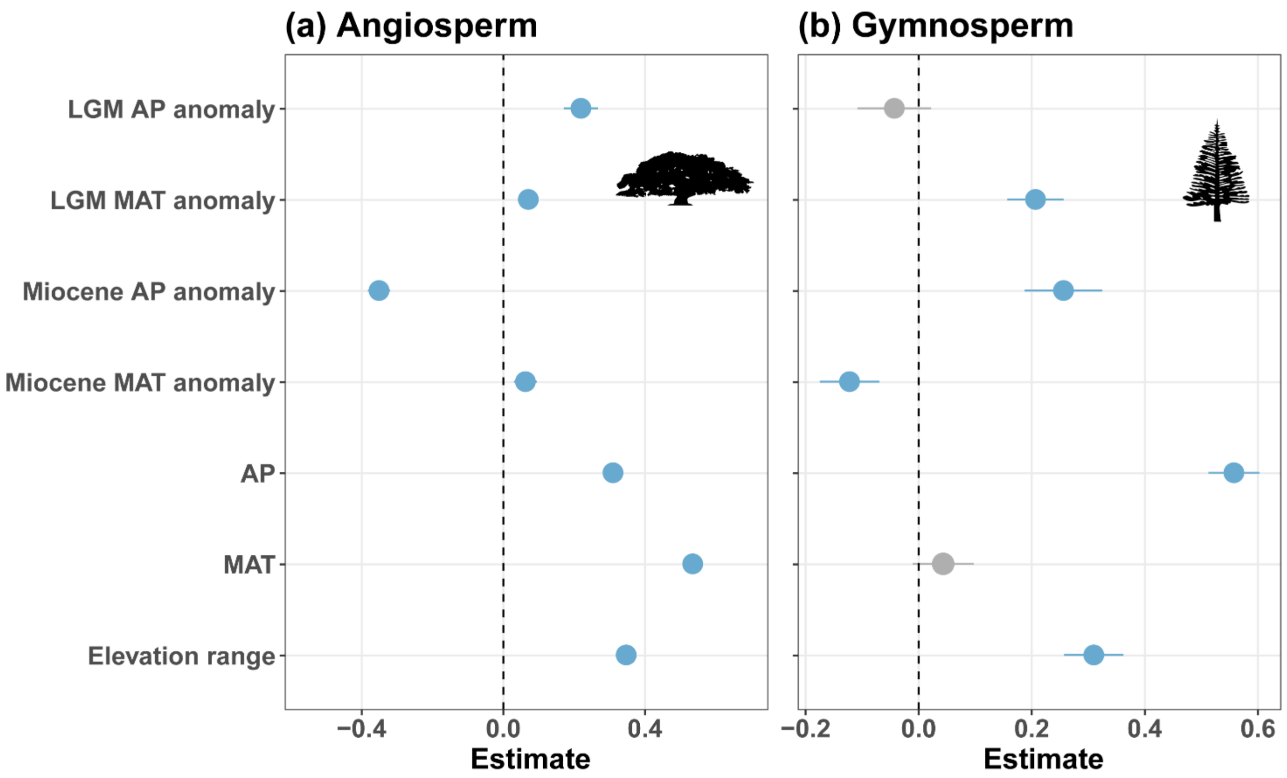

Fig. S4 Determinants of global phylogenetic endemism (PE) in (a) angiosperm and (b) gymnosperm trees. Estimates (standardized slopes) and 95% C.I.s were obtained from the non-spatial linear regression analyses. LGM, Last Glacial Maximum; MAT, mean annual temperature; AP, annual precipitation. Pictograms courtesy of PhyloPic (www.phylopic.org): (a) Tracy A. Heath; (b) T. Michael Keesey.

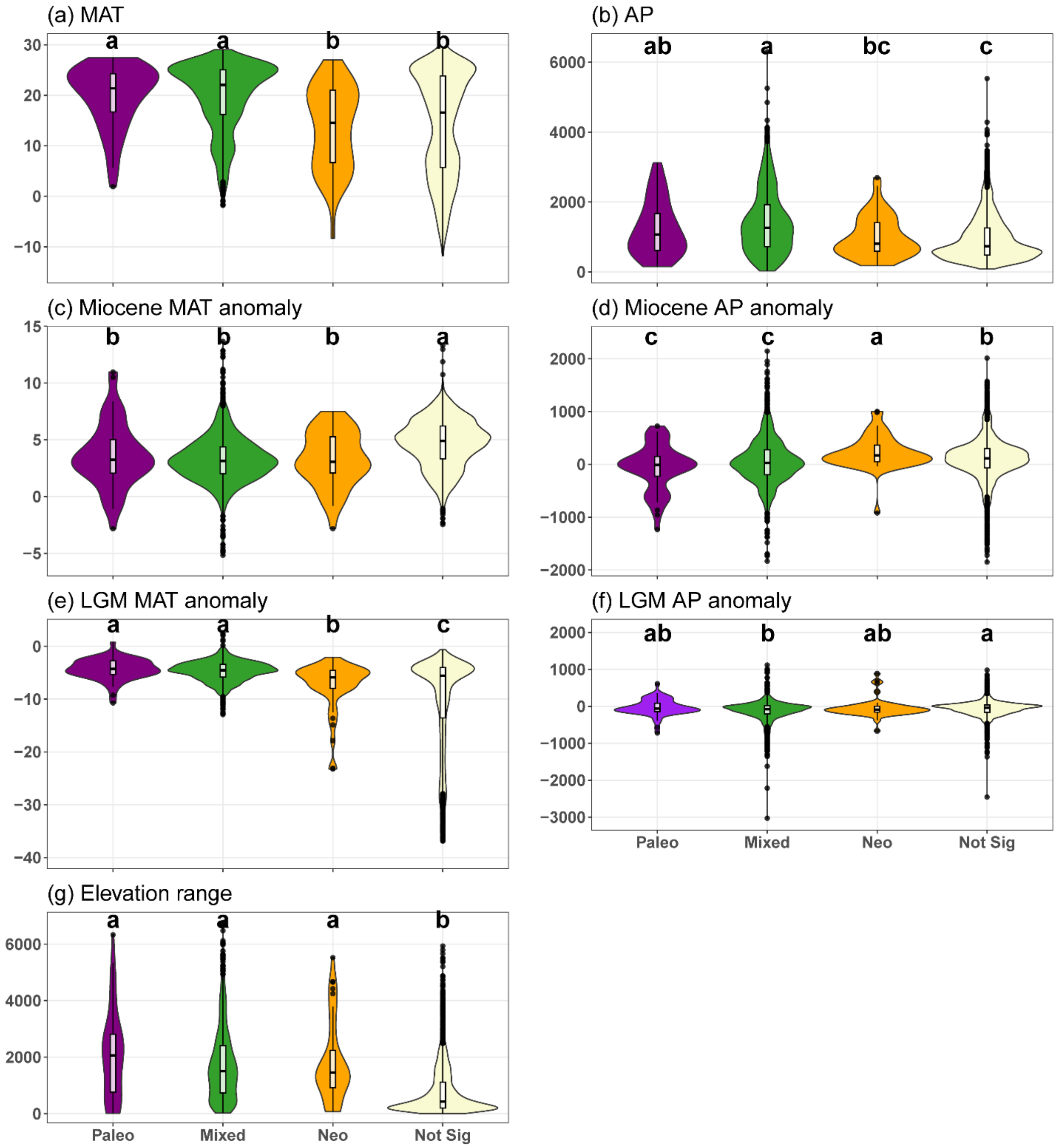

Fig. S5 Significance tests between each type of angiosperm endemism types (i.e., either neo-, paleo-, or mixed endemism as shown in Fig. 2a) and non-hotspots (non-significant regions in Fig. 2a) for each of the environmental variables. Significance test was carried out using the K-sample Fisher-Pitman permutation test (100,000 permutations) and the method “Tukey” for the multiple post-hoc tests. Different letters indicate significant differences among groups ($p < 0.05$ at least). LGM, Last Glacial Maximum; MAT, mean annual temperature; AP, annual precipitation

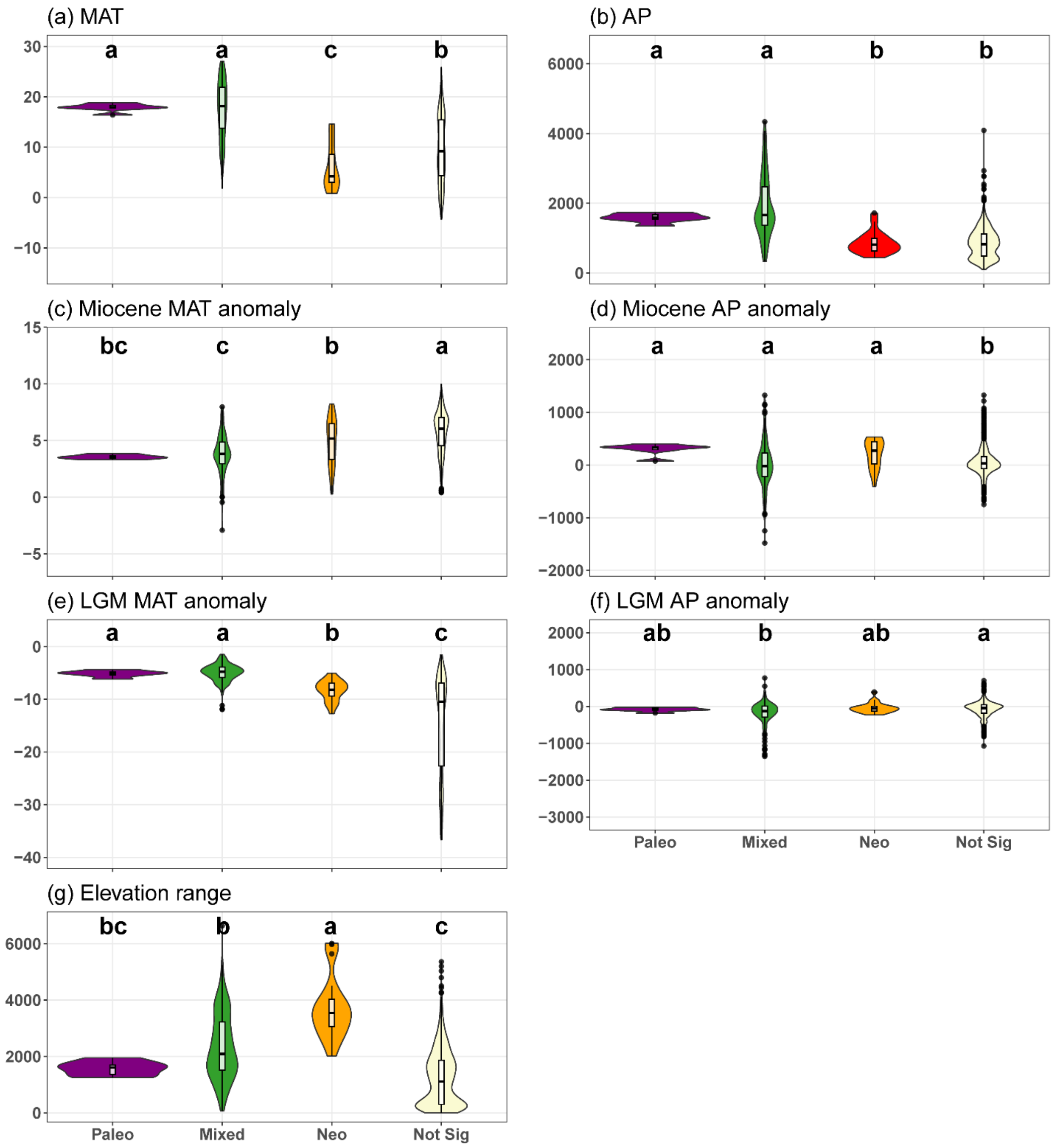

Fig. S6 Significance tests between each type of gymnosperm endemism types (i.e., either neo-, paleo-, or mixed endemism as shown in Fig. 2b) and non-hotspots (non-significant regions in Fig. 2b) for each of the environmental variables. Significance test was carried out using the K-sample Fisher-Pitman permutation test (100,000 permutations) and the method "Tukey" for the multiple post-hoc tests. Different letters indicate significant differences among groups ($p < 0.05$ at least). LGM, Last Glacial Maximum; MAT, mean annual temperature; AP, annual precipitation.

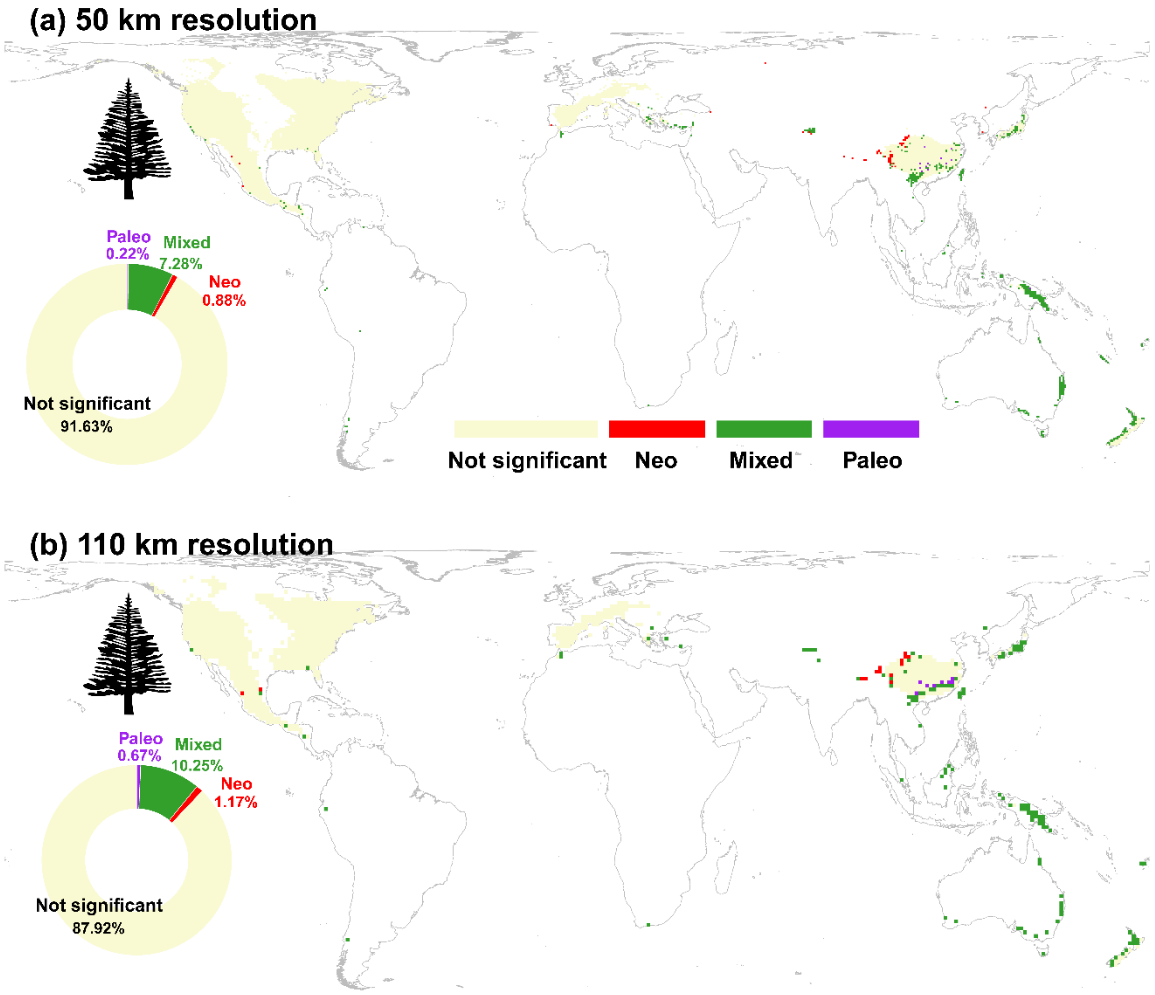

Fig. S7 Comparison of global distribution of gymnosperm tree endemism types. (a) and (b) each represents the result using a 50 × 50 km and a 100 × 100 km resolution. Centers of neo-endemism (Neo, i.e., concentrations of rare short branches), paleo-endemism (Paleo, i.e., concentrations of rare long branches), and mixed neo- and paleo-endemism (Mixed) were identified using a randomization analysis. Pictograms courtesy of PhyloPic (www.phylopic.org): T. Michael Keesey.

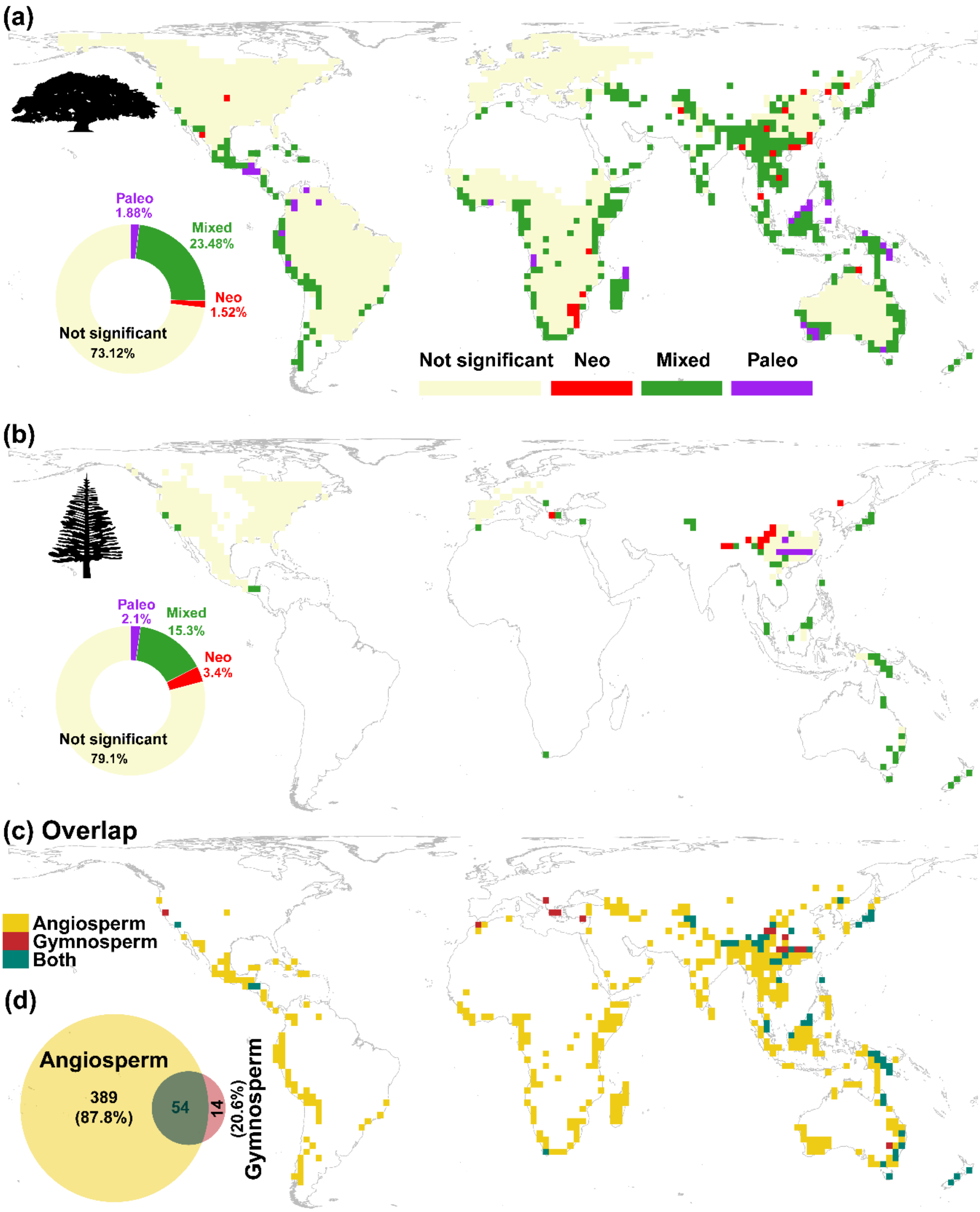

Fig. S8 Global distribution of tree endemism types for (a) angiosperm and (b) gymnosperm trees, and (c) their overlap, based on a 220 × 220 km resolution. Centers of neo-endemism (Neo, i.e., concentrations of rare short branches), paleo-endemism (Paleo, i.e., concentrations of rare long branches), and mixed neo- and paleo-endemism (Mixed) were identified using a randomization analysis [categorical analysis of neo- and paleo-endemism (CANAPE)] conducted separately for

angiosperm and gymnosperm trees. (d) Venn diagram showing the area and percentage overlap of the significant endemism regions (i.e., hotspot, including centers of either neo- endemism, paleo-endemism, or mixed neo- and paleo-endemism) between angiosperm and gymnosperm tree species. Numbers are sum of the hotspot cells, and the percentages in brackets correspond to the percentages of non-overlapping cells in each of the angiosperm and gymnosperm hotspots. Pictograms courtesy of PhyloPic (www.phylopic.org): (a) Tracy A. Heath; (b) T. Michael Keesey.

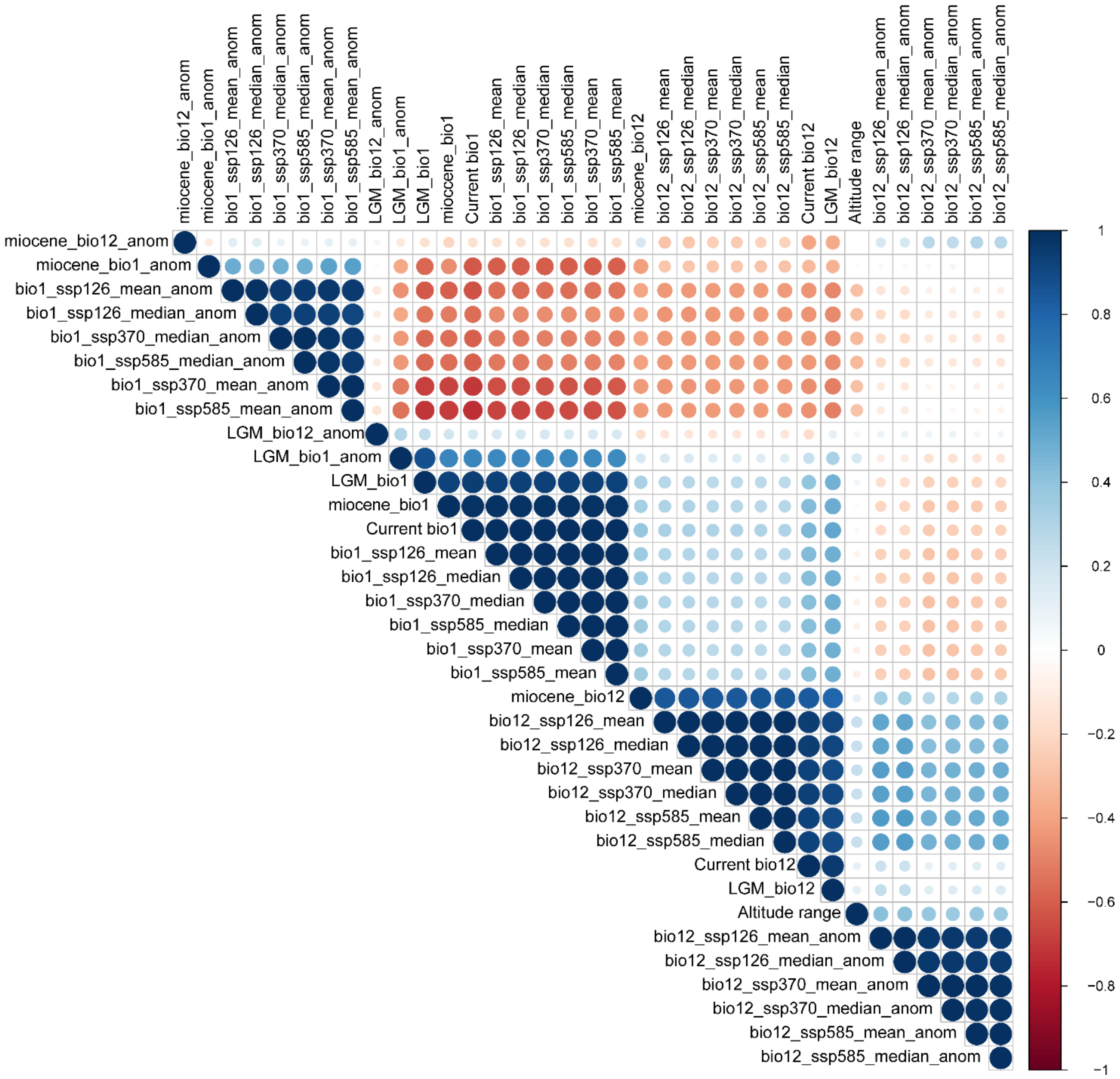

Fig. S9 Person's coefficient of all the environmental variables, including both paleoclimate and future climate and their anomalies.

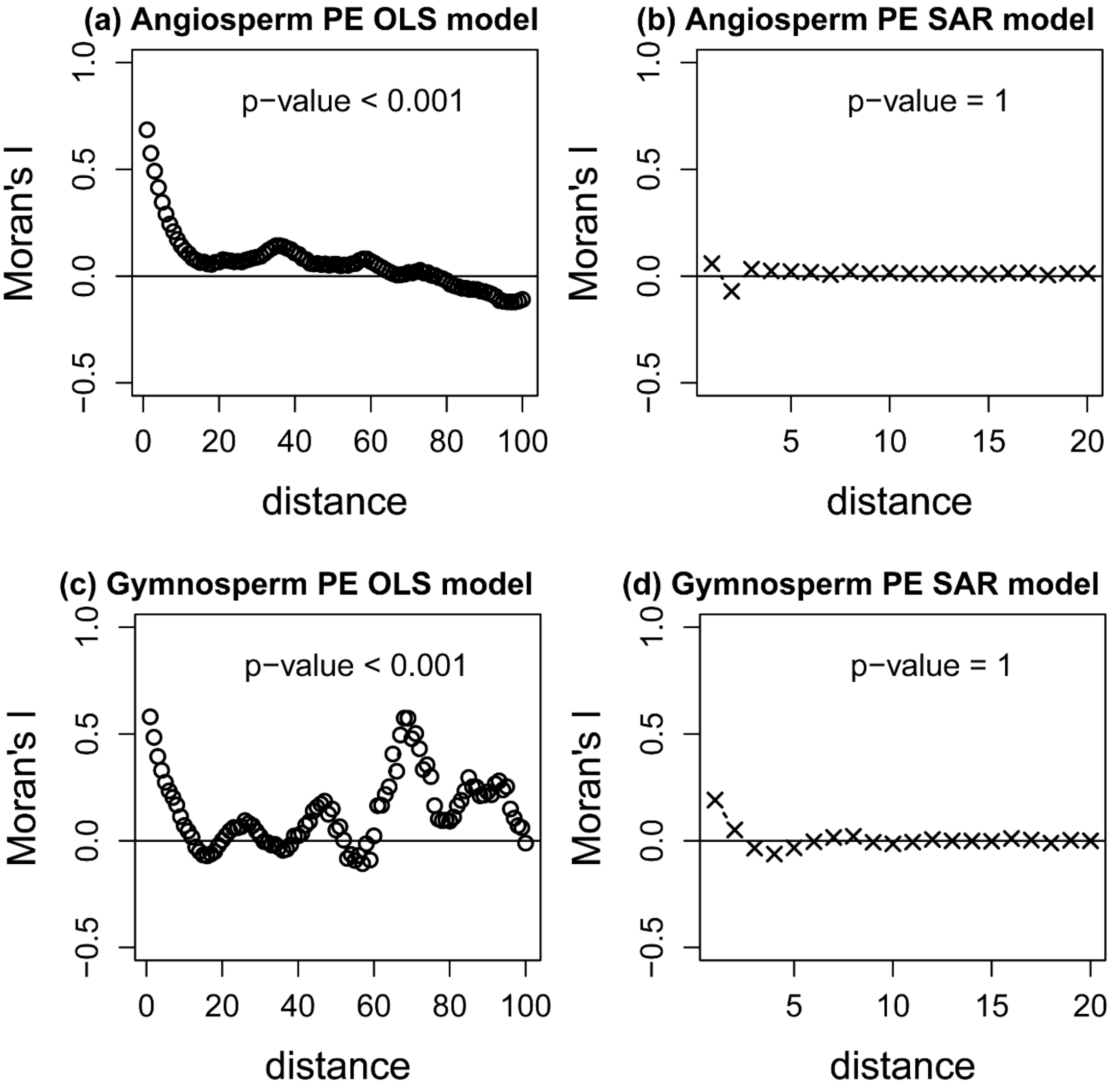

Fig. S10 Correlograms of Maron's *I* for (a & b) angiosperm and (c & d) gymnosperm tree phylogenetic endemism ordinary least squares (OLS) and simultaneous autoregressive (SAR) models, as shown in Figs. 3 & S5. The *p*-value in each subplot represents the significance of the Motan's *I* test, and significant *p*-value indicates the residual of the model has strong spatial autocorrelation, and vice versa.

Table S1 Environmental variables used in the study. **Time** only applies to climatic variables and is related to the time period represented by the variables. **Category** indicates the general climate situation in each time period or variable meanings (for the last two variables). Mya: Million years ago; kya: thousand years ago.

Variables	Time	Category	Original resolution	Source
Annual mean temperature (ºC)	Current climate (1970-2000)	Current	30 arc-seconds	WorldClim.org
Annual Precipitation (mm)	Current climate (1970-2000)	Current		WorldClim.org
Annual mean temperature (ºC)	Late Miocene climate (11.61-7.25 Mya)	Warm	2.5 arc-minutes	Pound et al., 2011
Annual precipitation (mm)	Late Miocene climate (11.61-7.25 Mya)	Warm		Pound et al., 2011
Annual mean temperature (ºC)	Last Glacial Maximum (*ca*. 21 kya)	Recent cold	30 arc-seconds	Karger et al., 2017 & chelsa-climate.org
Annual precipitation (mm)	Last Glacial Maximum (*ca*. 21 kya)	Recent cold		Karger et al., 2017 & chelsa-climate.org
Global human modification index	-	Human disturbance	30 arc-seconds	Kennedy et al., 2018
Elevation range (m)	-	Topographic heterogeneity	90 m	

Table S2 Details of climate models and scenarios used in future estimates (2071 – 2100) of climate anomaly. Data were extracted from Chelsa V2: CMIP6 scenarios.

**Models**	**Institute**
GFDL-ESM4	Geophysical Fluid Dynamics Laboratory, National Ocean and Atmospheric Administration, USA
IPSL-CM6A-LR	Institut Pierre-Simon Laplace, France
MPI-ESM1-2-HR	Max Planck Institut fur Meteorologie, Germany
MRI-ESM2-0	Meteorological Research Institute, Japan
UKESM1-0-LL	Met Office Hadley Centre, U.K.
**Scenarios**	
ssp126	SSP1-RCP2.6 climate as simulated by the GCMs
ssp370	SSP3-RCP7 climate as simulated by the GCMs
ssp585	SSP5-RCP8.5 climate as simulated by the GCMs

Table S3 Summary of the ordinary least squares (OLS) and simultaneous autoregressive (SAR) models for angiosperm species phylogenetic endemism. MAT: mean annual temperature; AP: Annual precipitation; LGM: Last Glacial Maximum.

**Linear model**									
Variable	**Estimate**	**SE**	**statistic**	**p.value**	**conf.low**	**conf.high**	**R^2**	**AIC**	**BIC**
Elevation range	0.346785	0.008151	42.54259	0	0.330806	0.362764	0.741	-2256.55	-2194.42
Miocene AP anomaly	-0.35109	0.016002	-21.9407	2.10E-103	-0.38246	-0.31972			
MAT	0.534542	0.009955	53.6933	0	0.515027	0.554058			
AP	0.309559	0.010697	28.93841	1.79E-174	0.288589	0.330528			
Miocene MAT anomaly	0.062247	0.016421	3.790687	0.000151	0.030057	0.094438			
LGM AP anomaly	0.218954	0.024572	8.910662	6.31E-19	0.170785	0.267122			
LGM MAT anomaly	0.070459	0.009893	7.122312	1.16E-12	0.051067	0.089852			
SAR model									
Variable	**Estimate**	**SE**	**statistic**	**p.value**	**conf.low**	**conf.high**	**R^2**	**AIC**	**BIC**
Elevation range	0.232608	0.010238	22.72106	0	0.212543	0.252674	0.904	-9501.88	-9432.85
Miocene AP anomaly	-0.06104	0.030621	-1.99334	0.046224	-0.12105	-0.00102			
MAT	0.257207	0.027192	9.459059	0	0.203912	0.310502			
AP	0.20908	0.022486	9.298024	0	0.165007	0.253152			
Miocene MAT anomaly	0.093296	0.027896	3.344469	0.000824	0.038622	0.147971			
LGM AP anomaly	0.164799	0.029933	5.50554	3.68E-08	0.106131	0.223467			
LGM MAT anomaly	0.175076	0.031954	5.478939	4.28E-08	0.112447	0.237705			

Table S4 Summary of the ordinary least squares (OLS) and simultaneous autoregressive (SAR) models for gymnosperm species phylogenetic endemism. MAT: mean annual temperature; AP: Annual precipitation; LGM: Last Glacial Maximum.

**Linear model**									
Variable	**Estimate**	**SE**	**statistic**	**p.value**	**conf.low**	**conf.high**	**R^2**	**AIC**	**BIC**
**Elevation range**	0.309632	0.026795	11.55552	1.23E-29	0.257072	0.362193	0.602	-29.1	18.67
Miocene AP anomaly	0.256079	0.034858	7.346352	3.35E-13	0.187703	0.324456			
**MAT**	0.0433	0.027528	1.572939	0.115946	-0.0107	0.097298			
**AP**	0.557397	0.022843	24.40115	7.56E-111	0.512589	0.602205			
**Miocene MAT anomaly**	-0.12228	0.026841	-4.55573	5.65E-06	-0.17493	-0.06963			
**LGM AP anomaly**	-0.0431	0.033126	-1.30117	0.193402	-0.10808	0.021876			
**LGM MAT anomaly**	0.206558	0.02536	8.145175	7.95E-16	0.156813	0.256302			
SAR model									
Variable	**Estimate**	**SE**	**statistic**	**p.value**	**conf.low**	**conf.high**	**R^2**	**AIC**	**BIC**
**Elevation range**	0.119366	0.029661	4.024407	5.71E-05	0.061232	0.177499	0.837	-1349.87	-1296.79
Miocene AP anomaly	-0.06258	0.046659	-1.34118	0.179861	-0.15403	0.028872			
**MAT**	0.035496	0.042374	0.837683	0.402209	-0.04756	0.118548			
**AP**	0.284449	0.042408	6.707473	1.98E-11	0.201332	0.367567			
**Miocene MAT anomaly**	-0.01177	0.029128	-0.40425	0.686029	-0.06886	0.045315			
**LGM AP anomaly**	0.247616	0.03639	6.804572	1.01E-11	0.176293	0.318938			
**LGM MAT anomaly**	0.133205	0.050115	2.657982	0.007861	0.034981	0.231429			
