# Supplementary material for: Climate change and land use threaten global hotspots of phylogenetic endemism for trees
Source: Nat Commun. 2023 Oct 31;14:6950. doi: 10.1038/s41467-023-42671-y (PMC10618213; doi:10.1038/s41467-023-42671-y)
Supplement: Supplementary file 2 — Reporting Summary [file 41467_2023_42671_MOESM2_ESM.pdf]

nature portfolio

Corresponding author(s): Wen-Yong Guo

Last updated by author(s): Oct 7, 2023

Reporting Summary

Nature Portfolio wishes to improve the reproducibility of the work that we publish. This form provides structure for consistency and transparency in reporting. For further information on Nature Portfolio policies, see our Editorial Policies and the Editorial Policy Checklist.

Statistics

For all statistical analyses, confirm that the following items are present in the figure legend, table legend, main text, or Methods section.

n/a	Confirmed	
☐	☒	The exact sample size (n) for each experimental group/condition, given as a discrete number and unit of measurement
☐	☒	A statement on whether measurements were taken from distinct samples or whether the same sample was measured repeatedly
☐	☒	The statistical test(s) used AND whether they are one- or two-sided<br>*Only common tests should be described solely by name; describe more complex techniques in the Methods section.*
☐	☒	A description of all covariates tested
☐	☒	A description of any assumptions or corrections, such as tests of normality and adjustment for multiple comparisons
☐	☒	A full description of the statistical parameters including central tendency (e.g. means) or other basic estimates (e.g. regression coefficient) AND variation (e.g. standard deviation) or associated estimates of uncertainty (e.g. confidence intervals)
☐	☒	For null hypothesis testing, the test statistic (e.g. F, t, r) with confidence intervals, effect sizes, degrees of freedom and P value noted<br>*Give P values as exact values whenever suitable.*
☒	☐	For Bayesian analysis, information on the choice of priors and Markov chain Monte Carlo settings
☒	☐	For hierarchical and complex designs, identification of the appropriate level for tests and full reporting of outcomes
☐	☒	Estimates of effect sizes (e.g. Cohen's d, Pearson's r), indicating how they were calculated

Our web collection on statistics for biologists contains articles on many of the points above.

Software and code

Policy information about availability of computer code

Data collection	Data used in the study came from several global databases which have been compiled by authors in previous work (https://github.com/wyeco/TC_conservation). The world tree species checklist (GlobalTreeSearch, GTS, v.1.6) was used to extract the global tree species list for the current study. The occurrence records of the selected species were collated from five widely used and publicly accessible databases, namely: the Global Biodiversity Information Facility (GBIF; the derived dataset summarizing the used occurrence records from 2,070 datasets in GBIF can be viewed and accessed via https://doi.org/10.15468/dd.4jvnmv), the public domain Botanical Information and Ecological Network v.3 (BIEN; http://bien.nceas.ucsb.edu/bien/), the Latin American Seasonally Dry Tropical Forest Floristic Network (DRYFLOR; http://www.dryflor.info/), the RAINBIO database (http://rainbio.cesab.org/), and the Atlas of Living Australia (ALA; http://www.ala.org.au/). The phylogeny was extracted using Smith & Brown (2018); environmental data were obtained from various sources, including CHELSA (https://chelsa-climate.org/), PaleoClim (http://www.paleoclim.org), Kennedy et al. (2019, Global Change Biology, 25: 811–826).
Data analysis	All R packages used in the data analysis are provided in the Methods section. Specifically, the "alphahull (v.2.2)" was used to construct alpha hulls; the "letsR (v.4.0)" package was used to rasterize the range maps; the "wdpar (v.1.3.4)" was used to extract the existing protected areas; the "spatialreg (v.1.2)" was used to run the SAR models; the "coin (v. 1.4-3) was used to run the permutation test. The R codes for the analyses are available on Github (https://github.com/wyeco/tree_PE_conservation) and mirrored on Zenodo (https://zenodo.org/record/8418763).

For manuscripts utilizing custom algorithms or software that are central to the research but not yet described in published literature, software must be made available to editors and reviewers. We strongly encourage code deposition in a community repository (e.g. GitHub). See the Nature Portfolio guidelines for submitting code & software for further information.

Data

Policy information about availability of data

All manuscripts must include a data availability statement. This statement should provide the following information, where applicable:

- Accession codes, unique identifiers, or web links for publicly available datasets
- A description of any restrictions on data availability
- For clinical datasets or third party data, please ensure that the statement adheres to our policy

The tree occurrence data are openly available from TREECHANGE (https://github.com/wyeco/TC_conservation). All other data used in the analyses were downloaded from public databases or papers: the phylogeny was extracted using Smith & Brown (2018); environmental data were obtained from various sources, including CHELSA (https://chelsa-climate.org/), PaleoClim (http://www.paleoclim.org), Kennedy et al. (2019, Global Change Biology, 25: 811–826). The data related to analyses are available on Github (https://github.com/wyeco/tree_PE_conservation) and mirrored on Zenodo (https://zenodo.org/record/8418763).

Research involving human participants, their data, or biological material

Policy information about studies with human participants or human data. See also policy information about sex, gender (identity/presentation), and sexual orientation and race, ethnicity and racism.

Reporting on sex and gender	N/A
Reporting on race, ethnicity, or other socially relevant groupings	N/A
Population characteristics	N/A
Recruitment	N/A
Ethics oversight	N/A

Note that full information on the approval of the study protocol must also be provided in the manuscript.

Field-specific reporting

Please select the one below that is the best fit for your research. If you are not sure, read the appropriate sections before making your selection.

☐ Life sciences ☐ Behavioural & social sciences ☒ Ecological, evolutionary & environmental sciences

For a reference copy of the document with all sections, see nature.com/documents/nr-reporting-summary-flat.pdf

Ecological, evolutionary & environmental sciences study design

All studies must disclose on these points even when the disclosure is negative.

Study description	We present global maps of tree species phylogenetic endemism, and describe their main environmental relations, especially the paleoclimate, using statistical tests (OLS and SAR). We further test the human pressure and future climate change pressure on those endemism centers, and their conservation status.
Research sample	Initial data collection was performed on all validated tree species (N = 41,835) following the GlobalTreeSearch v1.6, including taxonomic names, occurrence records, and phylogeny, from existing and openly accessible databases. In addition, environmental data were extracted either from openly accessible global high-resolution layers, or from the data creator with permission (e.g., the late Miocene climate).
Sampling strategy	The study uses all validated tree species with sufficient relevant data, such as occurrence records, and phylogenetic information.
Data collection	All distribution data were collected in 2017 by one of the main authors (Josep M. Serra-Diaz) with help of all other authors. The other data used in the analyses were obtained in 2022.
Timing and spatial scale	The spatial scale of the study is global, with a resolution of 110 km, although the original data resolution of much of the data is much finer (e.g., the CHELSA data are at a resolution of 30 arc-seconds [~1 km at the equator]). We further ran the main analyses using other resolutions (e.g., 220 km and 50 km) to validate the main findings.
Data exclusions	Due to the data quality and availability, we applied several exclusion steps in the study: 1) species name was not valid in the TNRS Taxonomic Name Resolution Service (http://tnrs.iplantcollaborative.org); 2) species occurrence was an outlier in environmental space, occurring in aquatic environments, urban areas, or botanical gardens; 3) selected occurrence records were not verified by species country checklists; 4) species was missing from the phylogeny; 5) island grid cells without environmental data were omitted from all statistical analyses.

Reproducibility	The study's data were obtained from various existing databases. The detailed data gathering and cleaning steps are already published (Serra-Diaz et al. 2017, Forest Ecosystems, 4: 30), and all occurrences used in the analysis are already deposited in BIEN (https://bien.nceas.ucsb.edu/bien/). In the Methods section, we have provided a detailed description of each analysis, such as R packages and set-ups, to make sure our analyses are reproducible. In addition, the custom code used in the study are available in a GitHub repository at https://github.com/wyeco/tree_PE_conservation and mirrored on Zenodo (https://zenodo.org/record/8418763).
Randomization	No experiment was conducted in the present study, as it is a global analyses.
Blinding	No experiment was conducted in the present study, as it is a global analyses.

Did the study involve field work? ☐ Yes ☒ No

Reporting for specific materials, systems and methods

We require information from authors about some types of materials, experimental systems and methods used in many studies. Here, indicate whether each material, system or method listed is relevant to your study. If you are not sure if a list item applies to your research, read the appropriate section before selecting a response.

Materials & experimental systems

n/a	Involved in the study
☒	☐ Antibodies
☒	☐ Eukaryotic cell lines
☒	☐ Palaeontology and archaeology
☒	☐ Animals and other organisms
☒	☐ Clinical data
☒	☐ Dual use research of concern
☒	☐ Plants

Methods

n/a	Involved in the study
☒	☐ ChIP-seq
☒	☐ Flow cytometry
☒	☐ MRI-based neuroimaging
